# Supplementary material for: Cryo-EM structures of the autoinhibited E. coli ATP synthase in three rotational states
Source: eLife. 2016 Dec 21;5:e21598. doi: 10.7554/eLife.21598 (PMC5214741; doi:10.7554/eLife.21598)
Supplement: Figure 2—source data 1. — DOI: http://dx.doi.org/10.7554/eLife.21598.005 [file elife-21598-fig2-data1.docx]

|  |  |  | State 1 | State 2 | State 3 |
| --- | --- | --- | --- | --- | --- |
| Molecular mass (kDa) | 558 | Particles | 95,345 | 67,829 | 53,587 |
| Sample support | Quantifoil R2/2 | Resolution (Å) | 6.9 | 7.8 | 8.5 |
| Microscope | Titan Krios | Running average frames/window | 7/19 | 7/19 | 7/19 |
| Detector | Falcon II | Accuracy rotations (°) | 3.12 | 3.37 | 3.8 |
| Voltage (kV) | 300 | Accuracy translations (pix) | 1.82 | 2.01 | 2.43 |
| Pixel size/Box size (Å) | 1.4/350 | Model-to-map correlation fit^ (across atoms in volume) | 0.92 | 0.92 | 0.92 |
| No. of movie frames | 20 |  |  |  |  |
| Exposure time (s) | 2 |  |  |  |  |
| Electron dose (e^-^. Å^2^) | 29 |  |  |  |  |
| Micrographs recorded/used | 8640/8388 |  |  |  |  |
| Particles picked/used | 395,140/  216,711 |  |  |  |  |

^ calculated using Chimera fit to map (Pettersen, E.F. et al. UCSF Chimera--a visualization system for exploratory research and analysis. *J Comput Chem* **25**, 1605-12 (2004).)
